# Wireless Remote Home Monitoring of Vital Signs in Patients Discharged Early After Esophagectomy: Observational Feasibility Study

**DOI:** 10.2196/21705

**Published:** 2020-12-04

**Authors:** Martine J M Breteler, Lieke Numan, Jelle P Ruurda, Richard van Hillegersberg, Sylvia van der Horst, Daan A J Dohmen, Mathilde C van Rossum, Cor J Kalkman

**Affiliations:** 1 Department of Anesthesiology University Medical Center Utrecht Utrecht Netherlands; 2 Luscii Healthtech BV Amsterdam Netherlands; 3 Department of Technical Medicine University of Twente Enschede Netherlands; 4 Department of Surgery University Medical Center Utrecht Utrecht Netherlands; 5 Biomedical Signals and Systems Group University of Twente Enschede Netherlands

**Keywords:** remote monitoring, wireless monitoring, vital signs monitoring, telemonitoring, wearables

## Abstract

**Background:**

Hospital stays after major surgery are shorter than ever before. Although enhanced recovery and early discharge have many benefits, some complications will now first manifest themselves in home settings. Remote patient monitoring with wearable sensors in the first days after hospital discharge may capture clinical deterioration earlier but is largely uncharted territory.

**Objective:**

This study aimed to assess the technical feasibility of patients, discharged after esophagectomy, being remotely monitored at home with a wireless patch sensor and the experiences of these patients. In addition, we determined whether observing vital signs with a wireless patch sensor influences clinical decision making.

**Methods:**

In an observational feasibility study, vital signs of patients were monitored with a wearable patch sensor (VitalPatch, VitalConnect Inc) during the first 7 days at home after esophagectomy and discharge from hospital. Vital signs trends were shared with the surgical team once a day, and they were asked to check the patient’s condition by phone each morning. Patient experiences were evaluated with a questionnaire, and technical feasibility was analyzed on a daily basis as the percentage of data loss and gap durations. In addition, the number of patients for whom a change in clinical decision was made based on the results of remote vital signs monitoring at home was assessed.

**Results:**

Patients (N=20) completed 7 days each of home monitoring with the wearable patch sensor. Each of the patients had good recovery at home, and remotely observed vital signs trends did not alter clinical decision making. Patients appreciated that surgeons checked their vital signs daily (mean 4.4/5) and were happy to be called by the surgical team each day (mean 4.5/5). Wearability of the patch was high (mean 4.4/5), and no reports of skin irritation were mentioned. Overall data loss of vital signs measurements at home was 25%; both data loss and gap duration varied considerably among patients.

**Conclusions:**

Remote monitoring of vital signs combined with telephone support from the surgical team was feasible and well perceived by all patients. Future studies need to evaluate the impact of home monitoring on patient outcome as well as the cost-effectiveness of this new approach.

## Introduction

Monitoring in high-care settings (eg, intensive care units) includes continuous measurement of different vital signs and frequent visual observations of the patient’s clinical status by the nurse. In low-care settings, such as surgical wards, the current standard is intermittent measurement of vital signs only, usually once every shift [1,2]. By contrast, when patients are discharged after major surgery, vital signs are no longer monitored at all, while it is known that more than 29% of deaths after noncardiac surgery occur after patients are discharged from the hospital [3]. Although the risk of patient deterioration has decreased by the time the patient is discharged from hospital, the risk that patient deterioration will go unnoticed increases.

At present, patients are discharged after major surgery earlier than ever before. In part, this is facilitated by the introduction of enhanced recovery after surgery programs that have shown to accelerate patient recovery, resulting in shorter hospital lengths of stay [4-6]. Although recovery within the patient’s own home has many benefits, it increases the risk that early warning signs will be missed; some late major complications might first manifest themselves in the home setting.

Recognizing the early signs of deterioration in the first few critical days at home might be improved with the availability of remote monitoring of vital signs for patients at high risk for complications, such as patients discharged home early after esophagectomy. Hospital readmissions after esophagectomy occur frequently, ranging from 5%-19%, and are associated with poor outcomes [4,7-9]. Advances in telemonitoring technology have now resulted in wearable and wireless sensors for remote unobtrusive vital signs monitoring. Such technology could provide patients the opportunity to recover at home, with the patient knowing that the hospital team will capture any possible deterioration early. At least in theory, this should allow safe early discharge after surgery and may reassure patients and their family.

Several studies have demonstrated the feasibility of wireless vital signs monitoring in patients admitted to the hospital [10-13], but monitoring patients at home in the first days after hospital discharge with wearable sensors is largely uncharted territory. It is unknown whether it is feasible to monitor patients remotely at home or whether remotely observing vital signs positively impacts clinical decision making.

Therefore, the objective of this study was to assess the technical feasibility of patients, discharged after esophagectomy, being remotely monitored with a wireless patch sensor as well as their experiences. In addition, we aimed to determine whether observing vital signs with a wireless patch sensor in these patients influenced clinical decision making.

## Methods

### Study Design and Setting

This was an observational feasibility study in which patients were monitored after esophagectomy with a wearable patch sensor (VitalPatch, VitalConnect Inc) on the general ward of the University Medical Center Utrecht, the Netherlands, and at home during the first 7 days after hospital discharge. The University Medical Center Utrecht ethics committee waived the need for formal ethical approval, since patients were not subject to procedures or required to follow extensive rules of behavior.

### Study Population

Patients receiving care after esophagectomy at the surgical oncology ward were included. Patients were recruited from July 2019 to December 2019. All patients were informed about the study 1 week before surgery by phone. Exclusion criteria were known skin allergies, pacemaker or implantable cardioverter defibrillator, or a wound near the application site of the patch. After written informed consent was obtained from the patient on the surgical ward, the wireless patch sensor was applied and vital signs recording started.

### Description of the Wireless Patch Sensor

The VitalPatch wearable biosensor consists of a disposable adhesive patch that incorporates 2 electrocardiography electrodes, a triaxial accelerometer, and a thermistor. It is designed to facilitate remote monitoring of patients on the ward as well as in the home setting after hospital discharge. Heart rate and respiratory rate measurements of a previous version of the VitalPatch sensor (HealthPatch, VitalConnect Inc) have been validated in high-risk patients in a clinical environment [14,15]. The patch can be applied on the patient’s chest, and it records heart rate, heart rate variability, respiratory rate, and skin temperature (every 4 seconds) and body posture and steps continuously (every second) for up to 5 days. Data were sent via Bluetooth to a mobile phone (Cubot King Kong 3, Shenzhen Huafurui Technology Co Ltd), which uploaded the data over cellular networks to the HealthStream (MedioBioSense Ltd) cloud platform. This app can display vital signs data in real time but was not designed to view long-term vital signs trends. Data could be stored for up to 18 hours on the sensor if connection between the patch sensor and mobile app was lost. Afterward, it would take half of the upload time of the live data to upload this offline data to the cloud platform. No identifiable patient information was entered on the mobile device or in the app to ensure compliance with European General Data Protection Regulations.

### Data Collection

Patients wore a patch sensor on the surgical ward and during the first 7 days after hospital discharge. In-hospital measurements were solely used to generate baseline data prior to discharge, and the patient’s vital signs were observed intermittently through care as usual. A new patch was applied upon discharge, and patients were taught how to replace a new patch after 5 days at home. In addition, they were instructed to keep the mobile phone charged and within a range of 10 m. It was made explicit that wearing a patch at home does not mean that the patient’s vital signs would be continuously observed. Instead, their vital signs trends were checked once every 24 hours.

**Figure 1:**
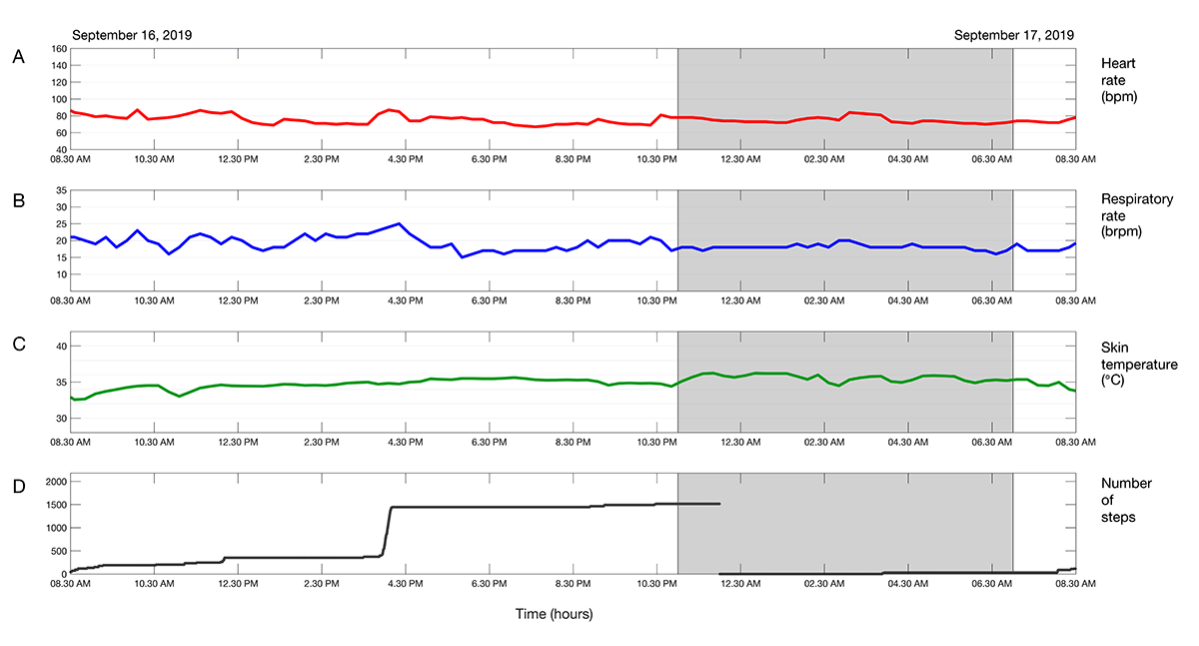
Example of vital sign trends over 24 hours, showing (A) heart rate, (B) respiratory rate, (C) skin temperature, and (D) cumulative step count. The shaded area indicates night-time.

**Figure 2:**
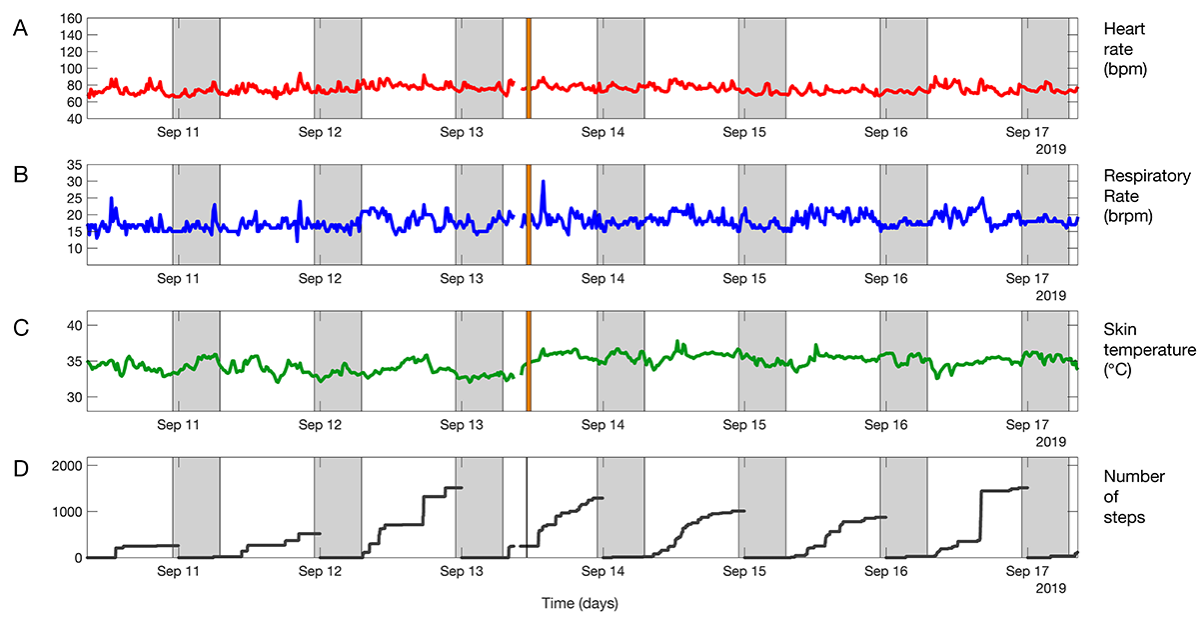
Example of vital sign trends over 7 days, both within hospital and at home after hospital discharge, showing (A) heart rate, (B) respiratory rate, (C) skin temperature, and (D) cumulative step count. The orange line indicates the time of hospital discharge. The shaded area indicates night-time.

Each morning, for 7 days postdischarge, vital sign trends over 24 hours and vital signs trends over 7 days were shared with the gastrointestinal oncology surgical team (3 surgeons, 2 surgical residents, 1 physician assistant) in a secure medical messaging app (Siilo, Siilo Holding BV). Examples are shown in Figure 1 and Figure 2. Surgeons were asked to check the patient’s condition each morning by phone using a short structured format with questions, such as “how do you feel?”, and asking about pain and fever. Phone calls were used as a safety net to prevent cases of missed patient deterioration, since the added value of remote vital signs monitoring had not been established. After each phone call, surgeons scored the patient’s condition with a 0 (no cause for concern), 1 (slightly worried), or 2 (significant concern). Conservative wait-and-see treatment was applied if a score of 1 was given, and the general practitioner was informed if a score of 2 was given. Thereafter, a surgical team member checked the vital sign trends and used that information to reassess their score. An *X* was scored if not enough vital signs data were available. This approach allowed the surgical team to adapt treatment policy, if needed, after taking into account information from the vital signs data trends.

### Signal Analysis

Wireless sensor data were retrieved in comma-separated variable (.csv) text files and stored in a secured local research database. Data reports were processed using Matlab (MathWorks Inc). A median filter over nonoverlapping epochs of 15 minutes was applied to eliminate artifacts from transients and to increase clarity and readability of the vital signs trend overviews. The number of steps was reset to zero at midnight to allow easy visual verification of each patient's daily activity level.

### Outcome Measures

Patient experiences of being remotely monitored at home and sensor wearability were assessed with a questionnaire, completed after the study. This questionnaire consisted of 8 questions using a 5-point Likert scale, 2 open answer questions, 1 yes or no question, and 1 question with 3 possible answers. The technical feasibility of remote home monitoring with a wireless sensor was assessed on a daily basis as the percentage of useful data available for vital signs interpretation. In addition, maximum duration of data loss was defined as gap durations less than 15 minutes, less than 1 hour, 1-4 hours, or 4 hours or longer. We distinguished data loss observed between the time of vital signs assessment (each morning) and observed at the end of the entire measurement period.

Another outcome measure was the number of patients in which a change in clinical decision was made based on the results of remote vital signs monitoring. This was measured by registering the number of times a score was adapted from 0 to 1, or from 1 to 2 following inspection of the vital signs trend overviews and compared with the check of the patient’s condition by phone each day. In addition, trend patterns of heart rate, respiratory rate, skin temperature, and number of steps during the week were also assessed.

### Statistical Analysis

Descriptive statistics were used to evaluate patient demographics and to assess feasibility of home monitoring. Since this was an observational feasibility study not designed to assess whether remote home monitoring could improve patient outcome, we refrained from a formal sample size calculation. Given the much lower probability of postdischarge adverse events [16], very large sample sizes would likely be needed to demonstrate statistically significant differences in outcome.

## Results

### Patient Population

Of 29 patients screened, 23 gave informed consent and 6 patients declined to participate, either because they already had “too much on their mind,” did not want to stay connected with the hospital once back home or thought they would not be able to cope with such modern technology. Two patients withdrew before the home monitoring period started because they were no longer willing to participate. One patient died during hospital admission. In total, 20 patients completed a combined total of 140 days (7 days each) of home monitoring with the wearable patch sensor. None of these patients were readmitted to the hospital within 30 days, and only 1 event after discharge home was observed (Table 1).

**Table 1:** Patient characteristics.

Characteristic		Patients (N=20)
Age (in years), median (IQR)		70 (7)
**Sex**		
	Male	16 (80)
	Female	4 (20)
BMI, median (IQR)		25 (2)
**Living status, n (%)**		
	Living alone	4 (20)
	Living with someone	16 (80)
**Comorbidities^a^, n (%)**		
	Hypertension	9 (45)
	Cardiovascular disease	5 (25)
	Chronic obstructive pulmonary disease	4 (20)
	Diabetes	3 (15)
Length of stay (days), median (IQR)		11 (7)
Readmission within 30 days, median (IQR)		0 (0)
**In-hospital postoperative events^a^, n (%)**		
	Pneumonia	13 (65)
	Atrial fibrillation	6 (30)
	Anastomotic leak	7 (35)
	Chyle leak	2 (10)
	Pneumothorax	1 (5)
	No events	2 (10)
**Postdischarge postoperative events, n (%)**		
	Severe dyspnea	1 (5)
	No events	19 (95)

^a^More than one event per patient possible; therefore, percentages do not add to 100%.

### Patient Experiences

Patient experiences were collected via a questionnaire as shown in Table 2. Overall, patients reported very high satisfaction rates. They appreciated that physicians checked their vital signs daily and they were happy to be called by the surgical team each day. The wearability of the patch sensor in the outpatient setting was high; patients were not aware of wearing a patch. Furthermore, no reports of skin irritation were mentioned, and the patch stayed in place most of the time, even during sweating and showering. One patient lost the patch twice at home, due to excessive sweating. Replacing the patch themselves at home was considered very easy. No information was visible on the dedicated mobile phone that acted as a gateway for the vital signs data, but patients were asked to keep the phone in close proximity to ensure uninterrupted data transmission. Interestingly, 95% of patients (19/20) reported they did not miss the absence of data on the mobile phone regarding current vital signs values or the proper functioning of the entire system. Thus, it did not bother them that they could not see anything on the mobile phone. Only 1 patient mentioned it would be reassuring to show the vital signs and additional information whether their vital signs data is being transferred to surgeons correctly. 75% of the patients (15/20) reported feeling safer at home knowing that their vital signs trends were checked and being called by a physician daily.

In addition, all patients were asked to imagine a future scenario in which they would be offered the option to go home 1 day earlier with a wireless patch sensor. Most patients (15/20, 75%) indicated they would prefer to be discharged earlier with the assistance of a remote patient monitoring solution. The main reasons given for this preference were a belief that they would recover more quickly at home and the fact that it is much more convenient to recover in one’s own home than in a hospital bed. A few patients mentioned that they felt quite uncertain being discharged home after such a major surgical procedure. As 1 patient noted: “It is quite a transition from a hospital where they constantly keep an eye on you, to home. It gives you reassurance when you have the feeling that your condition is being checked remotely.” Most of the patients reported the necessity of having home care properly organized, and ideally, having the possibility of access to home care 24 hours a day. Of note, the amount of home care received by these patients was dependent on their need for assistance with tube feeding or wound care. Three patients did not like the idea of being discharged sooner with assistance of a remote patient monitoring solution, either because they felt they were discharged quite quickly already (while they were still recovering from adverse events that occurred in-hospital) or they had experienced that their medications or home care was not adequately organized at the time they were discharged.

**Table 2:** Questions on patients’ experience of being remotely monitored at home with the VitalPatch sensor.

Question			Patient response (n=20)	
1	How did you experience wearing the patch in the first week after hospital discharge?^a^, mean			4.1
2	How did your partner experience the fact that you wore this patch and that your vitals were checked by physicians remotely?^a^, mean			4.5
3	To what extent did you find it pleasant or not pleasant that physicians were able to see your vital signs once daily?^a^, mean			4.4
4	To what extent did you find it pleasant or not pleasant that physicians called you each day to ask how you were doing?^a^, mean			4.5
5	**Nothing was visible on this mobile phone you had in proximity. Would you have preferred to see any data on this mobile phone, or you haven't missed this?, n (%)**			
		Yes		19 (95)
		No		1 (5)
6	**Your vitals were checked and you were called once daily. To what extent did this make you feel safer or not?, n (%)**			
		Yes		15 (75)
		No		5 (25)
7	**Imagine you have a choice to go home one day earlier with such a wireless patch sensor in the future. What do you think of this?, n (%)**			
		Yes		15 (75)
		No		5 (25)
8	What would you need for this, to make yourself comfortable at home? (open answer)			—^b^
9	To what extent were you aware of wearing this patch?^c^, mean			4.4
10	To what extent caused this patch irritation on your skin?^d^, mean			5
11	To what extent stayed this patch in place, even during sweating and showering?^a^, mean			4.8
12	To what extent was it easy to replace the patch at home?^a^, mean			4.8

^a^On a scale of 1 (disagree/disagreeable) to 5 (agree/agreeable).

^b^This was an open answer question.

^c^On a scale of 1 to 5, where a higher score indicates less awareness.

^d^On a scale of 1 to 5, where a higher score indicates less skin irritation.

### Feasibility of Home Monitoring

**Figure 3:**
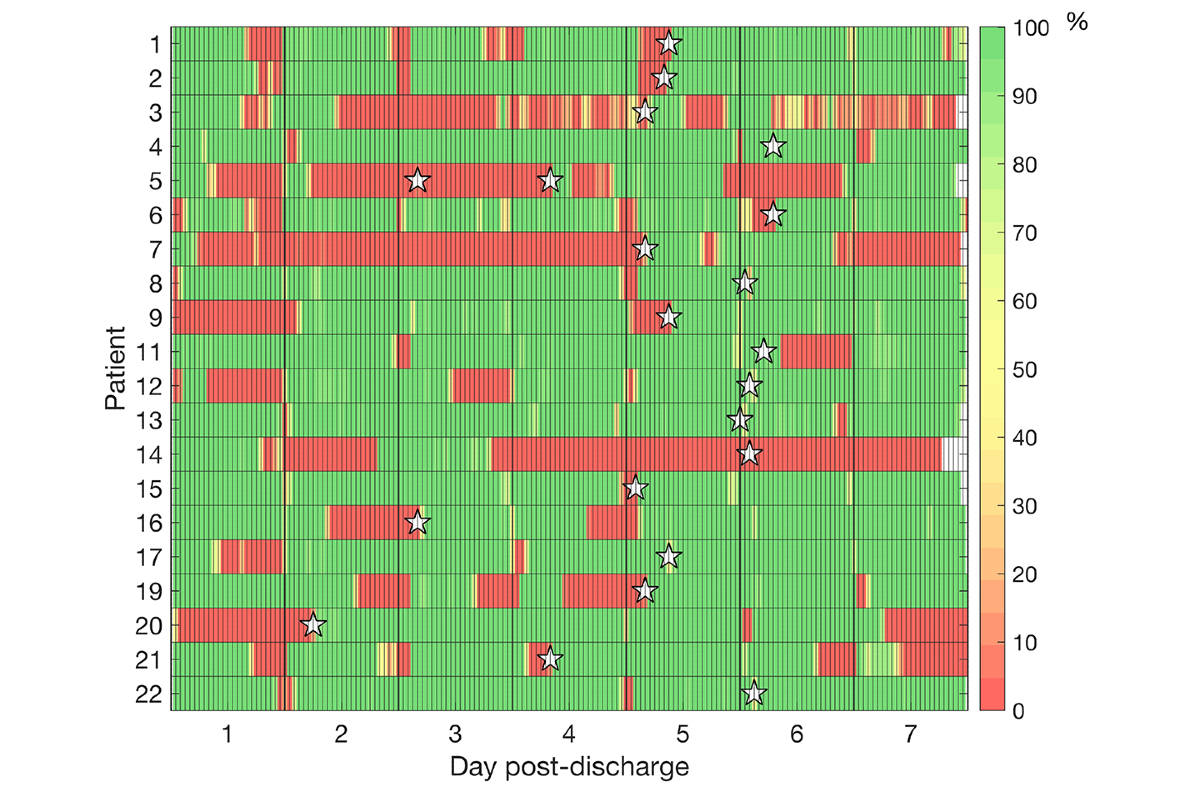
Percentage of available data (green) and data loss (red) of all patients per hour during each day of home measurements. Each star indicates the first measurement of a new patch.

Overall data loss of all vital signs at the time of assessment each morning and after the entire measurement period were a mean of 25% (SD 24%) and a mean of 14% (SD 19%), respectively. The amount of data loss varied considerably among patients as can be seen in Figure 3. At the time of patch replacement at home (by the patient themselves), most patients showed a preceding period with data loss. More than 77% of the gap durations at the time of vital signs assessment were less than 1 hour, with the majority of gaps lasting less than 15 minutes. An overview of frequency and duration of data loss is shown in Table 3.

**Table 3:** Amount of known data loss at the time of daily assessment (around 8:30 AM) and total amount of data loss as recorded at the end of the entire measurement period.

Type of data loss			Within previous 24 hours (observed at the time of assessment)		Within entire measurement period
Overall percentage, mean (SD)			25 (24)		14 (19)
**Gaps, n (%)**					
	<15 minutes	235 (55)		245 (67)	
	15-60 minutes	93 (22)		66 (18)	
	1-4 hours	66 (15)		37 (10)	
	>4 hours	35 (8)		19 (5)	

### Scoring of Vital Signs Trends in Patients at Home

**Figure 4:**
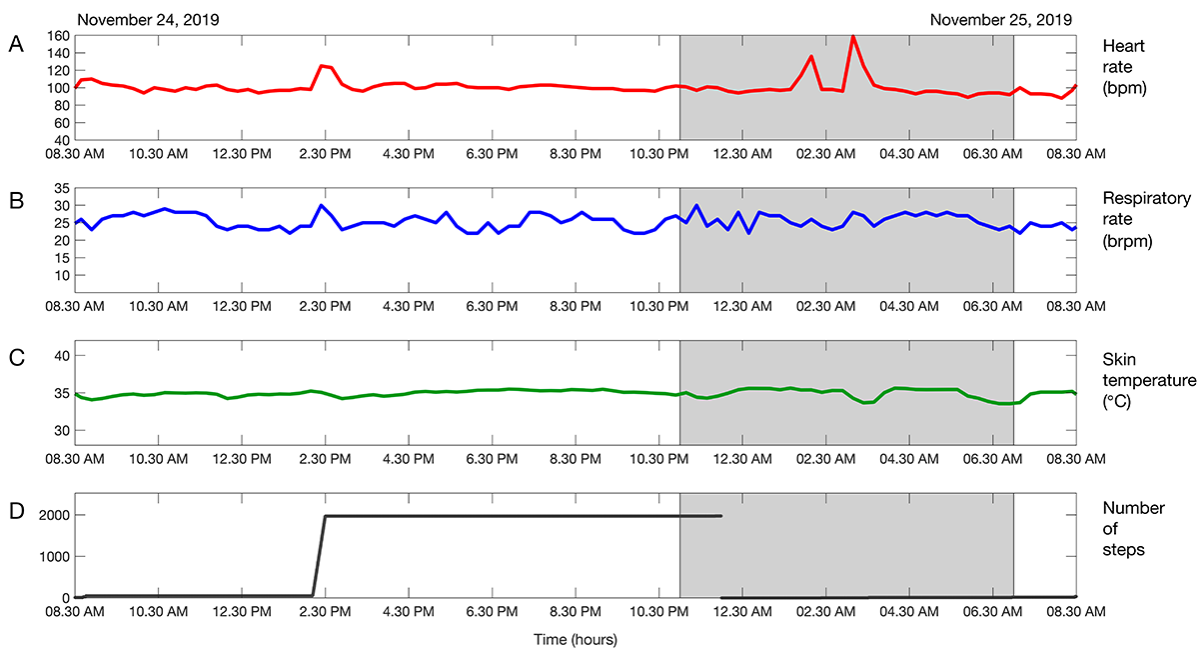
Vital sign trends over 24 hours, showing (A) heart rate, (B) respiratory rate, (C) skin temperature, and (D) cumulative step count of a patient who complained of severe dyspnea and coughing, when called at 8:30 AM (end of graph). Two episodes of increased heart rate can be seen during the night, but no clear vital signs deterioration occurred over the 24-hour period. The shaded area indicates night-time.

**Figure 5:**
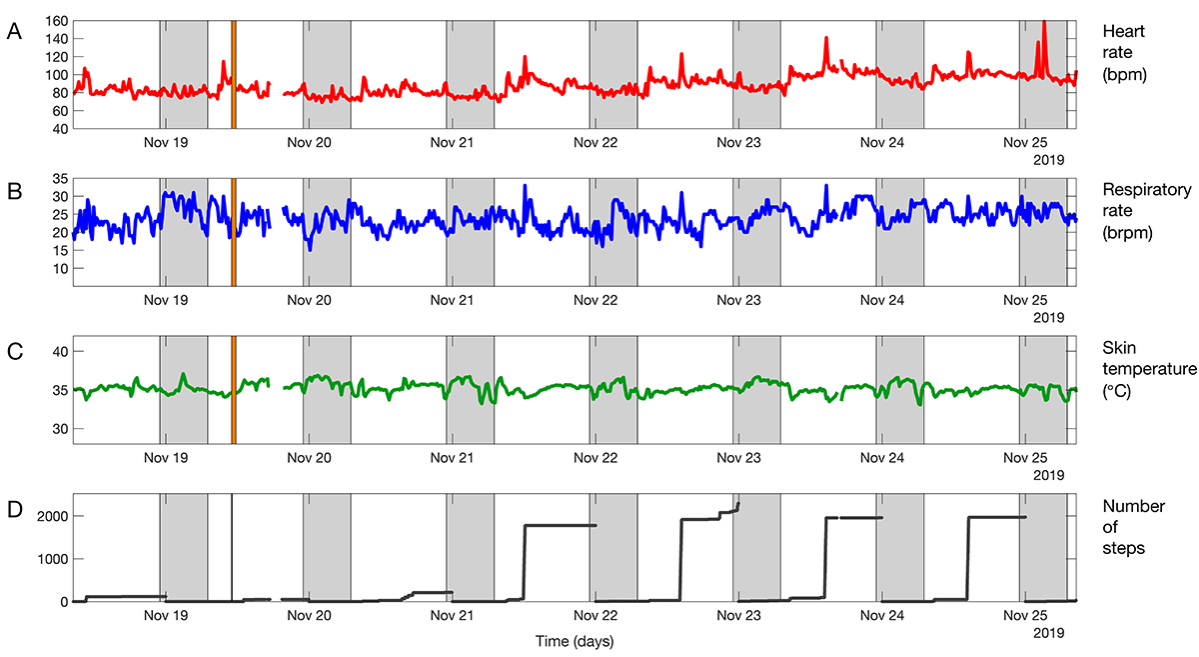
Vital sign trends over 7 days, showing (A) heart rate, (B) respiratory rate, (C) skin temperature, and (D) cumulative step count of the patient who complained of severe dyspnea and coughing on November 25. The orange line indicates the time of hospital discharge. Until November 22, heart rate fluctuated around 80 bpm at night and most respiratory rate values remained between 20 and 25 brpm. From November 22 until November 25, heart rate slowly increased from 80 to 100 bpm at night, while respiratory rate slightly increased to 25-30 brpm on November 23. The surgical team member asked the general practitioner to check the patient at home and prescribed bronchodilator treatment. The shaded area indicates night-time.

Table 4 shows an overview of scores provided after each call and vital signs observations. In 4/140 (3%) occasions, the surgeon was slightly worried about the patient’s condition after the phone call, but this did not result in an increased score after checking the vital signs trend overviews. As a result, clinical decision making was not changed based on observing vital signs. During 1 phone call the patient complained about severe dyspnea and coughing, after which a score of 2 (concern) was given and the general practitioner was asked to check on the patient’s condition and prescribed bronchodilator treatment. However, the vital signs trend overviews were not scored as worrisome (Figure 4 and Figure 5). Although no clear diagnosis could be found at this point in time, this patient continued struggling and was admitted to the hospital with atelectasis 4 weeks later. On 8/140 occasions (6%), a score of 1 (slightly worried) was assigned after checking the vital signs trends, most often related to a high heart rate at rest shortly after hospital discharge. Overviews of vital signs trends were not available on 9/140 (6%) occasions due to data loss.

**Table 4:** Overview of scores after phone calls and vital signs observations.

Observation		Value, n (%)
Phone calls		137 (98)
Missed calls		3 (2)
**Phone calls**		
	Slightly worried score, 1	4 (3)
	Concerned score, 2	1 (1)
**Vital signs observations**		
	Slightly worried score, 1	8 (6)
	Concerned score, 2	0 (0)
	Unable to judge, X	9 (6)

### Observing Vital Sign Trends Over Time

**Figure 6:**
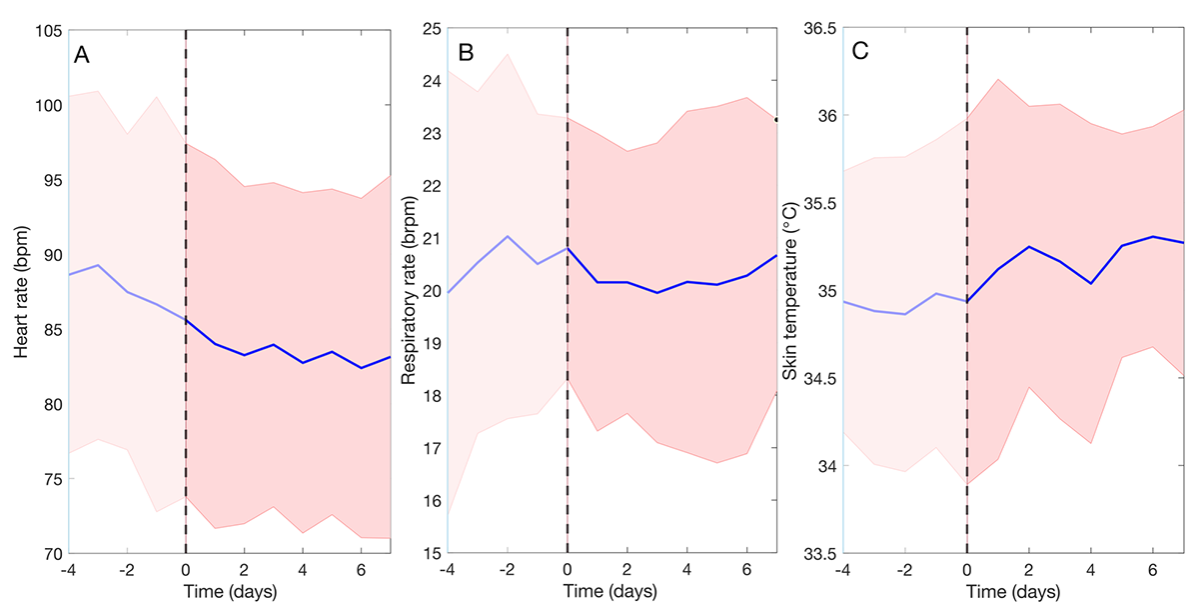
Mean (blue line) and SD (shaded red area) during night-time hours (for a period starting 4 days before hospital discharge until 7 days after discharge) of (A) heart rate, (B) respiratory rate, and (C) skin temperature of all patients.

**Figure 7:**
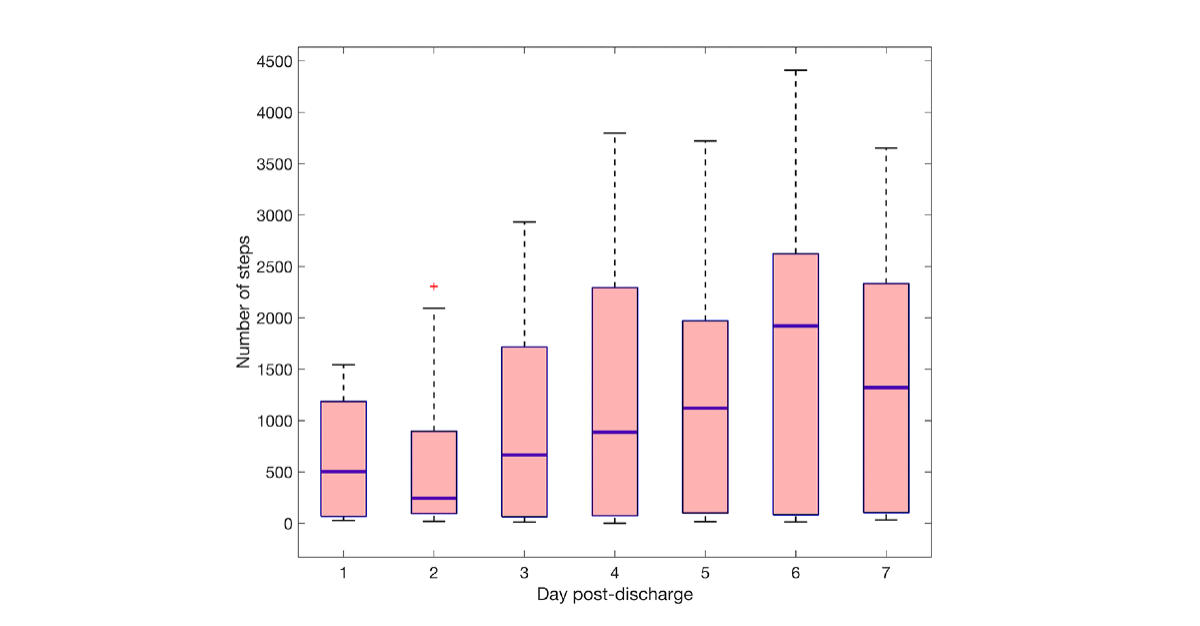
Box plots showing median daily number of steps for the first 7 days after hospital discharge.

Figure 6 provides an overview of the mean heart rate, respiratory rate, and skin temperature during night-time hours (11 PM to 7 AM) in the 4 days before hospital discharge until the first 7 days at home. Heart rate decreased from 89 bpm in-hospital to 85 bpm at home, whereas no change in respiratory rate was visible between the hospital and home period. Overall, high variation in heart rate and respiratory rate among patients at night could be seen. Skin temperature was slightly increased in the first days at home. Figure 7 shows a boxplot of the number of steps in the first 7 days after hospital discharge. The mean number of steps increased from 500 to 1300, suggesting that patients’ daily activity increases gradually as recovery progressed at home.

## Discussion

### Principal Findings

We investigated the feasibility of remote vital signs monitoring with a wireless patch sensor in patients after esophagectomy in the first week home after hospital discharge and assessed patient experiences. Each of the 20 patients who were monitored at home had a good recovery, and remotely observed vital signs trends did not directly alter clinical decision making, although it supported clinical judgments regarding the patients’ condition derived by the surgical team from the patient’s comments during the daily phone calls. In general, remote home monitoring was well perceived by patients and reported satisfaction scores and usability rates were very high. For the sensor used in this study, average data loss of vital signs measurements at home was 25%; both data loss and duration of data gaps varied considerably among patients. In this select group of patients recovering from major surgery, we observed a decrease in heart rate and an increase in number of steps during the first 7 days at home.

### Strengths and Limitations

When interpreting the findings of this study, some limitations should be taken into account. Based on previous studies, we had anticipated a 10% readmission rate in patients after esophagectomy [9,17]. However, we observed only 1 event after discharge at home, and none of the 20 study participants were readmitted to the hospital. Only much larger studies can demonstrate how vital signs trend patterns vary among patients with and without clinical deterioration after hospital discharge. As a result, we were unable to determine whether observing vital signs trends remotely changed clinical decision making. However, we cannot entirely eliminate the possibility that patients who decided to participate in this study had a better baseline prognosis or that a Hawthorne effect—the awareness of being observed remotely and daily phone contact with the surgical team—had positively influenced study outcomes [18,19].

A second limitation was our inability to discern the causes of the positive patient experiences. Both the fact that the patient’s vital signs were remotely checked and their daily telephone contact with a surgical team member might have contributed. In any case, patients highly appreciated being remotely monitored at home and having daily contact with the team, and as a result, they felt more reassured. Studies have shown that structured telephone calls following discharge can reduce readmission rates in elderly patients [20]. Although these findings cannot be translated to the our study, it seems likely that the ability to communicate with a patient to verify the presence of any deteriorating symptoms, together with abnormal vital signs, may improve recognition of patient deterioration in the home setting.

A score of 1 (slightly worried) was assigned in 6% of the vital signs trend reviews (8/140), most often related to a higher heart rate, especially shortly after hospital discharge or due to high respiratory rates. Elevated heart rates—possibly related to the process of recovery and postoperative fatigue—have been noticed in an earlier study after major surgery [21]. We observed that average heart rate slightly decreased in the days following discharge home. In contrast, average respiratory rate remained high in these patients monitored at home. One possible explanation could be technical in nature since the measurement approach in this particular sensor tends to overestimate respiratory rate. In a previous study [14,15], we validated a precursor of the VitalPatch sensor in surgical patients and observed considerable overestimation of respiratory rate. Carefully performed validation studies in clinical practice are therefore of crucial importance for a sustainable long-term implementation of remote wireless monitoring [22].

Nobody knows how often one should measure a full set of vital signs in patients discharged after major surgery. The VitalPatch sensor used in this study measures each of the vital signs in a nearly continuous (every 4 seconds) fashion. This seems redundant for measuring patients in a home setting who are no longer at high risk for deterioration and may increase the rate of false alarms. In addition, transmitting these continuous data streams consumes valuable energy and may easily contribute to data loss. In this study, 35/429 (8%) of the data gaps were longer than 4 hours which may result in difficulties interpreting vital signs trends appropriately. Reasons for data gaps may be the fact that the VitalPatch relies on Bluetooth technology and a smartphone acting as a gateway between the patch and remote medical server. Patients did not always remember to keep the phone in close proximity, for example during the night-time. Furthermore, they may have forgotten to keep the phone charged all the time, which may not always have been reported to us. Other reasons might be the inability to automatically restore connection with the cloud server after Bluetooth disconnection occurred or to transfer piled amounts of data after repetitive periods of connection loss. The high number of long duration data gaps is possibly related to the data transmission protocol of the mobile app used in this study. Data could be stored for 18 hours on the patch sensor if Bluetooth connection was transiently lost, but it took an additional 50% of time on top of the upload time of the live data to transfer this offline data to the cloud platform. Although this data transmission protocol could be improved, it is unknown to what extent the duration of such data gaps results in the inability to capture clinical deterioration on time in the home setting. However, it seems likely that a reduced monitoring frequency might be a necessary trade-off to minimize the number of alerts due to missing data. As soon as an alert is generated, a dedicated 24/7 medical call center could initiate video communication, for example, to verify the presence of any signs that might give reason for rapid medical attention and exclude trivial causes for the alert such as exercise. These approaches are especially relevant since the majority of patients at home will not deteriorate but may develop unimportant vital signs abnormalities which should not trigger intervention.

### Comparison With Prior Work

Studies that evaluate the feasibility and patient experiences of remote home monitoring are limited. A recent study of Tonino et al [23] demonstrated high wearability and good usability of the VitalPatch sensor worn by a small number of patients in the outpatient setting receiving blood cell transfusions or immunotherapy. The results of our study confirm these findings. Another study [24] reported high wearing comfort of the HealthPatch sensor in senior participants after long-term monitoring of 50 days in their home setting. Although the study [24] hints at the convenience of wireless monitoring of patients in the home care setting, the results were obtained in healthy volunteers and may therefore differ when used in patients discharged home.

Despite the fact that hospital-to-home initiatives are still in its infancy, the increasing pressure from payers force hospitals to develop less expensive alternatives to hospital care. A recent randomized controlled trial [25] compared direct costs of acute care in patients admitted to an emergency department, who were randomized to either usual hospital care or hospital-at-home care while vital signs were continuously monitored via the HealthPatch sensor. Although the sample, with 20 patients, was small in size and recruited within a highly selected patient group, the authors found that patients who received hospital-at-home care were readmitted less frequently within 30 days (7% vs 23%), and their health care costs were 38% lower on average. Nonetheless, large well-controlled studies in patients at risk for deterioration are needed to evaluate the impact of remote monitoring on patient outcomes.

### Conclusions

A daily 24-hour vital signs trend evaluation combined with a phone call from the surgical team were feasible and highly appreciated by all patients. The minimal requirements regarding optimal measurement frequency and data continuity for adequate home monitoring need to be further investigated. Remote patient monitoring at home is feasible. Future studies need to evaluate the impact of home monitoring on patient outcome as well as the cost-effectiveness of this approach.
